# Parkinsonism and dysautonomia with anti-CV2/CRMP5 associated paraneoplastic neurological syndromes mimicking multiple system atrophy: a case report

**DOI:** 10.1186/s12883-021-02448-6

**Published:** 2021-10-26

**Authors:** Jia Song, Ying Zhang, Yue Lang, Yi-Heng Wang, Jie Shao, Li Cui

**Affiliations:** Department of Neurology, Jilin University First Hospital, Xinmin Street 1, Changchun, Jilin China

**Keywords:** Paraneoplastic syndrome, Anti-CV2 antibody, Multiple system atrophy, Collapsin response mediated protein 5, Dysautonomia

## Abstract

**Background:**

Paraneoplastic neurological syndromes (PNSs) are broad-spectrum disorders that can affect any part of the nervous system varying in core symptoms. Onconeural antibodies, including Hu, Yo, Ri, anti-CV2, amphiphysin, Ma2, and Tr are well-characterized and commonly used for the diagnosis of definite PNS. Generally, anti-CV2 antibodies have usually been associated with cerebellar ataxia, chorea, peripheral and autonomic neuropathies, myelopathy, optic neuritis, and retinitis. However, Parkinsonism has not been reported as the core symptom in patients with anti-CV2 antibodies.

**Case presentation:**

We report a patient with anti-CV2 antibody manifested as Parkinsonism and autonomic dysfunction, which may lead to the diagnosis of multiple system atrophy with predominant Parkinsonism (MSA-P). A lumbar puncture examination was undergone to find a positive anti-CV2 antibody in cerebrospinal fluid. PET-CT showed no tumor. Immunotherapy was adopted and the symptoms were relieved for 5 months. However, with no evidence of tumor, he died after 8 months.

**Conclusions:**

Our findings indicate that PNS with anti-CV2 antibody can be shown as MSA-P mimic. Considering that MSA is a neurodegenerative disease with a poor prognosis, screening for other treatable or controllable factors like PNS presented in this case is necessary when encountering a rapid progressive MSA-mimic patient.

**Supplementary Information:**

The online version contains supplementary material available at 10.1186/s12883-021-02448-6.

## Background

PNS represents the remote effects of tumors on the nervous system, which is not associated with invasion or compression of the tumor but by activation of the immune system. Antibodies produced by the body against the tumor antigen may damage the nervous system. These antibodies are divided into 1) antibodies against neuronal nuclear and cytoplasmic antigens and 2) antibodies against cell surface and synaptic proteins. As a member of the former first identified as a biomarker of PNS in 2001 [1], collapsin response mediated protein 5 (CRMP5) is a neuronal cytoplasmic protein recognized by anti-CV2 antibody expressed in the cerebellum, hippocampus, thalamus, peripheral neurons, spinal cord, retina, and in SCLCs [1, 2]. Accordingly, the clinical presentations associated with anti-CV2 antibody include cerebellar ataxia, chorea, peripheral and autonomic neuropathies, myelopathy, optic neuritis, and retinitis [1, 3, 4]. Anti-CV2 antibody related PNS is closely related to small cell lung cancer and thymoma [1, 3]. The symptoms can be significantly alleviated after tumor-targeted treatment. Here, we report a case with anti-CV2 associated PNS mimicking MSA without tumor. It reminds physicians of the possibilities for diseases that present as MSA, especially tumor related PNS.

## Case presentation

A 70-year-old man presented to hospital with a 6-month history of slow movements with leaning forward and postural instability. Erectile dysfunction also occurred for more than half a year, before the motor symptoms. A month ago, the symptoms worsened. Meanwhile, he suffered from severe dizziness while standing, constipation and urinary incontinence in this month.. He was a heavy smoker previously without subjective loss of smell, seizures, or changes in body weight. He had no diabetes, family history of genetic diseases or exposure to toxic substances.

His supine blood pressure (BP) was 106/76 mmHg with heart rate (HR) 82 beats/ min, while it could not be measured accurately when standing. He showed hypophonia and reduced facial expression. The muscle strength score in extremities was 5/5. He also showed bradykinesia, bilateral reduced arm swing and right upper limbs and both lower limbs rigidity without tremor. The deep tendon reflexes were normal and plantar reflex was flexor bilaterally.

**Fig. 1:**
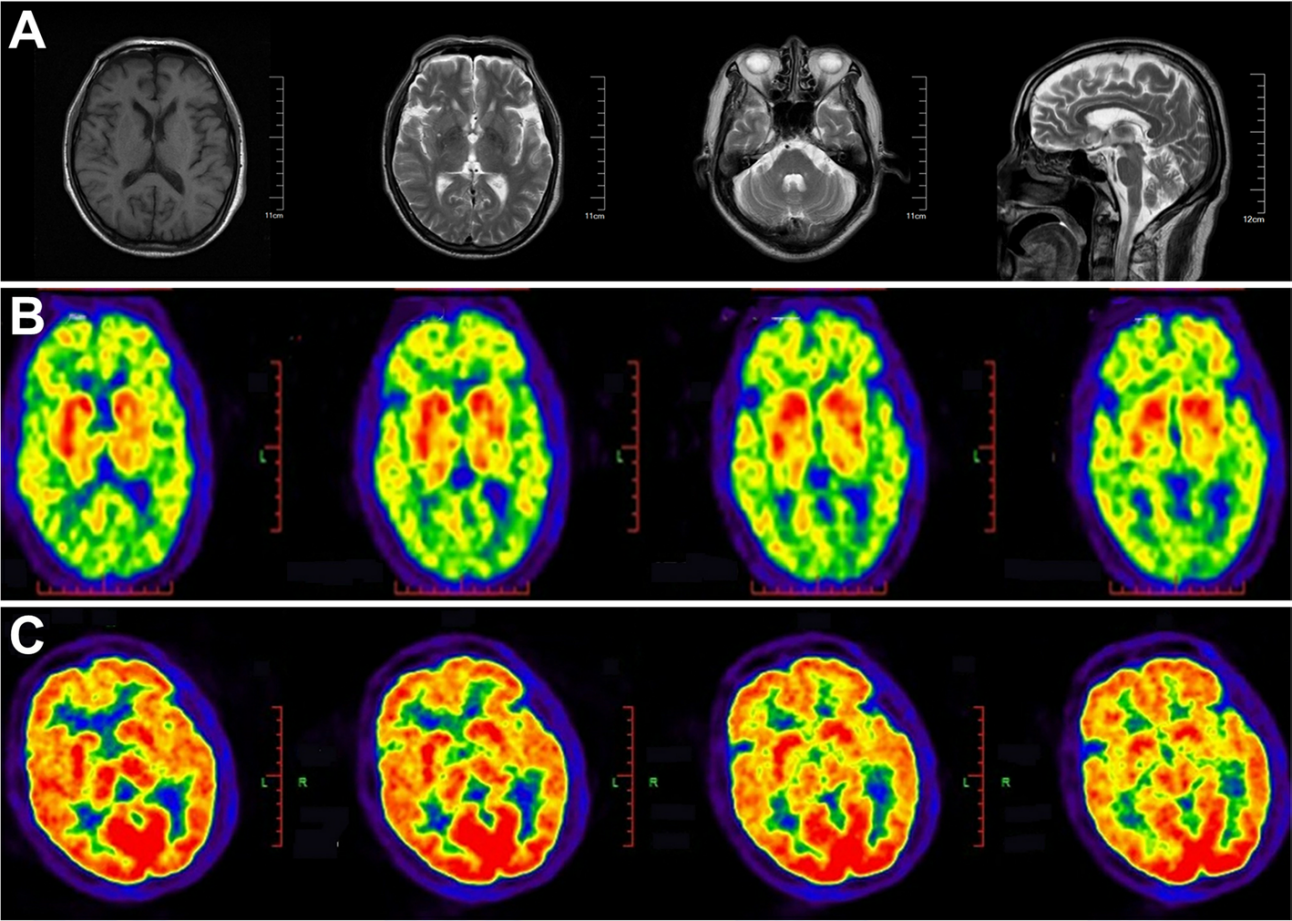
**A** Images of the patient’s MRI. Brain MRI showed multiple ischemic foci without brain stem or cerebellar atrophy. **B** Images of the patient’s ^11^C-CFTPET/CT. ^11^C-CFTPET/CT scan showed low metabolism of the left caudate nucleus and bilateral putamen. **C** Images of the patient’s ^18^F-FDGPET/CT. ^18^F-FDGPET/CT scan showed symmetrical low metabolism of bilateral frontal-parietal lobes

The values of thyroid function test, tumor markers were within the normal range. Results of serum anti-HIV antibody and hepatitis C antibody were negative. Urinary ultrasound showed 130 mL of post-void residual urine. Midbrain sonography and thymus computed tomography (CT) were normal. Multi-detector row CT scan of the lungs showed nodules with no thickening of bronchial wall, enlargement of hilar and mediastinal lymph nodes or atelectasis. Brain magnetic resonance imaging (MRI) showed no brain stem or cerebellar atrophy (Fig. 1A). The 11C-CFTPET/CT scan showed reduced uptake of 11C-CFT in the left caudate nucleus and bilateral putamen (Fig. 1B), and 18F-FDGPET/CT scan showed symmetrical hypometabolism of bilateral frontal-parietal lobes (Fig. 1C).

Based on his clinical information, he developed dysautonomia accompanied by Parkinsonism, which led to the diagnosis of MSA-P. According to the second consensus statement on the diagnosis of multiple system atrophy, he met the criteria of probable MSA [5]. Considering his hypotension, levodopa-benserazide 12.5 mg /3.125 mg were given orally three times a day. A week later, the dose was doubled. However, he showed no improvement in bradykinesia and instability posture, and the stiffness in the limbs prevented him from moving around at will. What’s worse, he suffered from a severe drop in BP, which set him free from a levodopa trial. Meanwhile, he was recommended to wear elastic socks, raise head 30° when lying, use abdominal straps when standing and drink adequate water. At the same time, droxidopa 100 mg was given orally once a day. However, his BP did not improve significantly. In case of supine hypertension, the dose was not increased and midodrine was not used.

Meanwhile, his dysautonomia progressed relatively rapidly. There was no supportive imaging evidence for MSA. Considering that MSA is a neurodegenerative disorder with no effective treatment and poor prognosis, we checked for other treatable or controllable factors. He underwent a lumbar puncture examination. In the cerebrospinal fluid (CSF), protein was 460 mg / L and total leukocyte count was 4.00 × 10^6^ / L. The anti-CV2 antibody was positive in the CSF and negative in the serum. Then a whole-body PET-CT scan was done and no tumor was detected. Based on the recommended diagnostic criteria for PNS [3], we diagnosed the patient as definite PNS with the nonclassical neurological syndrome, well-characterized onconeural antibodies, and no cancer.

Intravenous immunoglobulin was given at 0.4 g/kg/day. After 5 days of immunotherapy, his dizziness was significantly improved and he could walk independently. His supine BP was 130/83 mmHg with HR 79 beats/min. When standing, his BP was 123/75 mmHg with HR 82 beats/min at 1 min and 115/66 mmHg with HR 85 beats/min at 3 min. The BP was higher than that on admission with the absence of orthostatic hypotension. He received active hydration and medication every day since admission. However, the BP did not improve until the use of immunoglobulin, suggesting that it was the immunotherapy that worked. Moreover, there was no post-void residual urine in reexamination. As the symptoms relieved, intravenous immunoglobulin was stopped after 10 days. In consideration of the age and side effects, immunotherapy was refused by his relatives in the follow-up therapy. Considering that neurological symptoms may appear before the discovery of tumor in some PNS patients, we a followed him up dynamically.

**Fig. 2:**
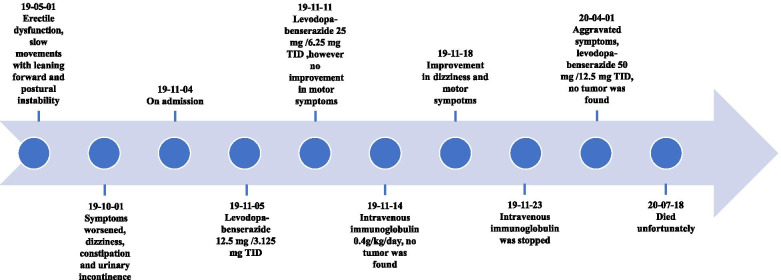
Clinical manifestations and treatment options organized as a timeline. The clinical manifestations and treatment options was consolidated as a timeline

Five months later, his symptoms worsened characterized by frequent falls, inability to walk independently, dysphagia and unintelligible speech. His limbs were too tense to be bent. Likewise, no positive results were found in tumor screening. The BP was 140/89 mmHg, and levodopa-benserazide 50 mg /12.5 mg were given orally three times a day for months. However, it did not work. Unfortunately, he died after 8 months. We also organized the patient’s clinical manifestations and treatment options as a timeline (Fig. 2).

## Discussion and conclusions

The patient’s clinical presentation was classified as “non-classical neurological syndrome”, indeed, the presentations of dysautonomia and Parkinsonism in anti-CV2 antibody related PNS are rare. Although autonomic neuropathy is taken as common signs, only seven patients showed Parkinsonism without severe and rapidly progressive dysautonomia [1, 6–9]. Here, we summarized the clinical characteristics of these 7 patients in tabular form (Additional Table 1).

Among the 7 patients (excluding this case), the ratio of male: female was approximately equal (42.9%). The mean age of onset was 60.3 (range 45-72 years old) during the 4 patients whose ages were recorded. Clinical manifestations were described in detail in 4 patients, with concomitant symptoms including: dysarthria (*n* = 1,25%) hoarse voice (*n* = 1,25%), autonomic nerve damage (*n* = 2,50%, one constipation, the other urinary urgency), sleep disturbance (*n* = 1, 25%, core symptom), and loss of taste (*n* = 1,25%). Basal ganglia changes were found in all the 4 patients (100%) whose brain MRI results were described, which could account for Parkinsonism. One case was combined with the thalamus and brainstem changes (25%). Two cases of MRS examination showed a reduction of NAA peak located in bilateral basal ganglia (*n* = 1,50%) and bilateral lateral ventricles (*n* = 1,50%). Lumbar puncture results were shown in 4 cases, 2 patients had elevated CSF protein levels (50%), with a maximum CSF protein of 629 mg/L. Three patients underwent EEG examination, and 2 showed abnormal results (66.7%) with slow waves in the temporal region. Four cases described the sites of antibody in detection, 2 in both blood and cerebrospinal fluid (50%), and 2 merely in blood (50%). Four cases gave the information about complicated tumor, treatment and prognosis. Two patients suffered from SCLC (50%), and radiotherapy (1 case, 50%) and chemotherapy (2 cases, 100%) were adopted to perform clinical improvements. One patient diagnosed with breast cancer (25%) got improvements in life quality after surgery. No tumor was found in one patient (25%) who received hormone treatment: dexamethasone 10 mg for 3 days and 15 mg for 30 days by intravenous drip, then 6 mg orally once a day with 0.75 mg reduced every 7 days. During the following up, the patient suffered from originally uncontrollable tremors and new onset of seizure. In all, patients achieved cancer treatment when tumors were found are more related to better outcomes than patients accessed to immunotherapy with no tumor.

Here, we report the first case of anti-CV2 antibody-positive PNS mimicking MSA-P. We speculate antibodies damage CRMP5 distributed in the basal ganglia and autonomic nervous system. However, the mechanism remains unknown. PNS treatments consist of tumor-targeted therapy, immunotherapy, and symptomatic treatment. Immunotherapy is indicated in patients with positive anti-CV2 antibody acting as pain, gross or fine motor skills, impairment in speech function. However, in general, immunotherapy is not effective in PNS patients with intracellular antibodies such as anti-CV2 antibody [4]. Instead, the symptoms can be significantly alleviated after targeted treatment. The prognosis of PNS has a great relationship with the type of primary tumor and onconeural antibody. Dubey et al. [4] found that patients with CRMP5 have a better 5-year survival rate than patients with ANNA1.

In addition to PNS, many diseases exhibit Parkinsonism and dysautonomia simultaneously (Additional Table 2). Physicians should make a comprehensive differential diagnosis as follows especially treatable diseases: 1) Neurodegenerative diseases: MSA-P, Parkinson’s Disease, Dementia with Lewy bodies. They are mostly shown as slow onset with old age and rare family history. Parkinsonism and autonomic dysautonomia can occur with different core symptoms. Neuroimaging shows characteristic features. There is no effective treatment with poor prognosis, so it is necessary to exclude other treatable diseases. 2) Hereditary degeneration diseases: fragile X-associated tremor ataxia syndrome (FXTAS), spinocerebellar ataxia (SCA), Perry syndrome. SCA and Perry syndrome are autosomal dominant, while FXTAS X chromosome dominant. The patients suffering from hereditary degeneration diseases are younger than those of neurodegenerative diseases with slow progression. Cerebellar ataxia often acts as the core symptom and some patients may display Parkinsonism and autonomic dysfunction. Genetic testing is reliable. Likewise, there is no effective treatment requiring to be carefully identified. 3) Hereditary metabolic diseases: Wilson’s disease, Gaucher’s disease-related to Parkinsonism. They are genetically related disorders at a young age and slow onset of abnormal cellular metabolism inherited as an autosomal recessive pattern. Wilson’s disease is a copper accumulation disorder, while Gaucher’s disease is a lysosomal metabolism disorder. Multiple organs are involved, and the nervous system tends to be one part. Genetic or laboratory testing can help with the confirmation of diagnosis. As for treatment, dietary changes and replacement therapy with enzymes have a positive effect on patients. We need to take this kind of disease into consideration as the treatment can alleviate the symptoms. 4) Infection and autoimmune diseases: Sjogren’s syndrome, acquired immune deficiency syndrome [10]. Patients have histories of infection or autoimmune diseases. Symptoms can deteriorate rapidly. Etiological examination or pathological biopsy is probative for diagnosis. It has to be emphasized that immunotherapy leads to a better prognosis, thereby the possibility of such diseases should be considered preferentially. 5) PNS [11]. Older patients showed rapid progression and risk factors associated with tumors. Generally, clinical manifestations are closely related to the type of antibody. Examination of paraneoplastic antibodies in the blood and CSF and the screening for tumors is required. Treatment aiming at tumors can relieve symptoms. Even with no tumor found, immunotherapy can relieve symptoms for some time as is described in this report. Therefore, we should actively detect PNS-related antibodies and the presence of primary tumors in patients with MSA symptoms. 6) Systemic light chain amyloidosis [12]. It’s a rare disease caused by extracellular deposition of amyloid with rapid progression. Symptoms are related to organs where amyloid is deposited (such as the heart, liver, kidney, and peripheral nerves) with a case report showing MSA-mimic. Tissue biopsy or abdominal liposuction is convincing. Patients are commonly treated with chemotherapy, but the prognosis is generally poor for organ complications. Given that, it is of great significance to check for treatable factors.

In this study, we report a case of anti-CV-2-associated Parkinsonism and rapidly progressive dysautonomia. The characteristics are as follows: 1) PNS patients with anti-CV-2 antibody have been reported to be associated with Parkinsonism (Additional Table 1), yet no dysautonomia or mild dysautonomia were described in these cases. Here, we reported a patient associated with anti-CV-2 antibody with rapid progression (compared with MSA-P patients) of severe autonomic nervous symptom impairment and Parkinsonism, which is difficult to differentiate from MSA-P in clinical manifestations. We speculate that CRMP5 is expressed in both autonomic nervous system and basal ganglia, as evidenced by reduced uptake of 11C-CFT in the left caudate nucleus. 2) Tumor-specific treatment is beneficial to clinical outcomes in patients with PNS. However, our patient was repeatedly screened for tumors to show negative results, and immunotherapy is a valuable treatment for temporary relief of symptoms. Therefore, on the one hand, screening for tumors should be carried out throughout the course of the disease, on the other hand, immunotherapy can be taken as an alternative as early as possible when no tumor is found.

However, there are limitations in this case: 1) Considering the absence of evidence of tumors and economic reasons, the patient did not recheck PET-CT and the antibody after immunotherapy and during his follow-up. 2) Autopsy was not performed after death, so the exact cause of death could not be determined, nor could it be determined whether the patients had antigen-antibody complex deposition and pathological changes in basal ganglia and autonomic nervous system in the brain tissue, so as to explore the mechanism of anti-CV-2 antibody in cerebrospinal fluid causing Parkinsonism and autonomic nervous dysfunction. 3) Due to limited conditions, the titer of anti-CV-2 antibody in cerebrospinal fluid and the specific binding ratio of dopamine were unknown. This study reminds physicians of the differential diagnosis of MSA-P, especially PNS when encountering patients with Parkinsonism and rapidly progressing autonomic failure.

## Supplementary Information

**Additional file 1: Table 1.** Clinical characteristics of anti-CV-2 related PNS presenting with Parkinsonism.

**Additional file 2: Table 2.** Summary of diseases manifested as Parkinsonism and dysautonomia.

## Data Availability

Data sharing is not applicable to this article as no datasets were generated or analyzed during the current study.

## Abbreviations

AIDS: Acquired immune deficiency syndrome
BP: Blood pressure
CRMP5: Collapsin response mediated protein 5
CSF: Cerebrospinal fluid
CT: Computed tomography
DLB: Dementia with Lewy bodies
ED: Erectile dysfunction
EEG: Electroencephalography
FDG PET: Fluorodeoxyglucose positron emission tomography
FLAIR: Fluid-attenuated inversion recovery
FXTAS: Fragile X-associated tremor ataxia syndrome
HIV: Human immunodeficiency virus
HR: Heart rate
MIBG: Meta-iodobenzylguanidine
MRI: Magnetic resonance imaging
MRS: Magnetic resonance spectroscopy
MSA-P: Multiple system atrophy predominant Parkinsonism
NAA: N-acetylaspartate
OH: Orthostatic hypotension
PD: Parkinson’s disease
PET: Positron emission tomography
PNS: Paraneoplastic neurological syndromes
REM: Rapid eye movement
SCA: Spinocerebellar ataxia
SCLC: Small-cell lung carcinoma
